# Protein asparagine deamidation prediction based on structures with machine learning methods

**DOI:** 10.1371/journal.pone.0181347

**Published:** 2017-07-21

**Authors:** Lei Jia, Yaxiong Sun

**Affiliations:** Amgen Inc., One Amgen Center Drive, Thousand Oaks, CA, United States of America; UMR-S1134, INSERM, Université Paris Diderot, INTS, FRANCE

## Abstract

Chemical stability is a major concern in the development of protein therapeutics due to its impact on both efficacy and safety. Protein “hotspots” are amino acid residues that are subject to various chemical modifications, including deamidation, isomerization, glycosylation, oxidation etc. A more accurate prediction method for potential hotspot residues would allow their elimination or reduction as early as possible in the drug discovery process. In this work, we focus on prediction models for asparagine (Asn) deamidation. Sequence-based prediction method simply identifies the NG motif (amino acid asparagine followed by a glycine) to be liable to deamidation. It still dominates deamidation evaluation process in most pharmaceutical setup due to its convenience. However, the simple sequence-based method is less accurate and often causes over-engineering a protein. We introduce structure-based prediction models by mining available experimental and structural data of deamidated proteins. Our training set contains 194 Asn residues from 25 proteins that all have available high-resolution crystal structures. Experimentally measured deamidation half-life of Asn in penta-peptides as well as 3D structure-based properties, such as solvent exposure, crystallographic B-factors, local secondary structure and dihedral angles etc., were used to train prediction models with several machine learning algorithms. The prediction tools were cross-validated as well as tested with an external test data set. The random forest model had high enrichment in ranking deamidated residues higher than non-deamidated residues while effectively eliminated false positive predictions. It is possible that such quantitative protein structure–function relationship tools can also be applied to other protein hotspot predictions. In addition, we extensively discussed metrics being used to evaluate the performance of predicting unbalanced data sets such as the deamidation case.

## Introduction

**Fig 1:**
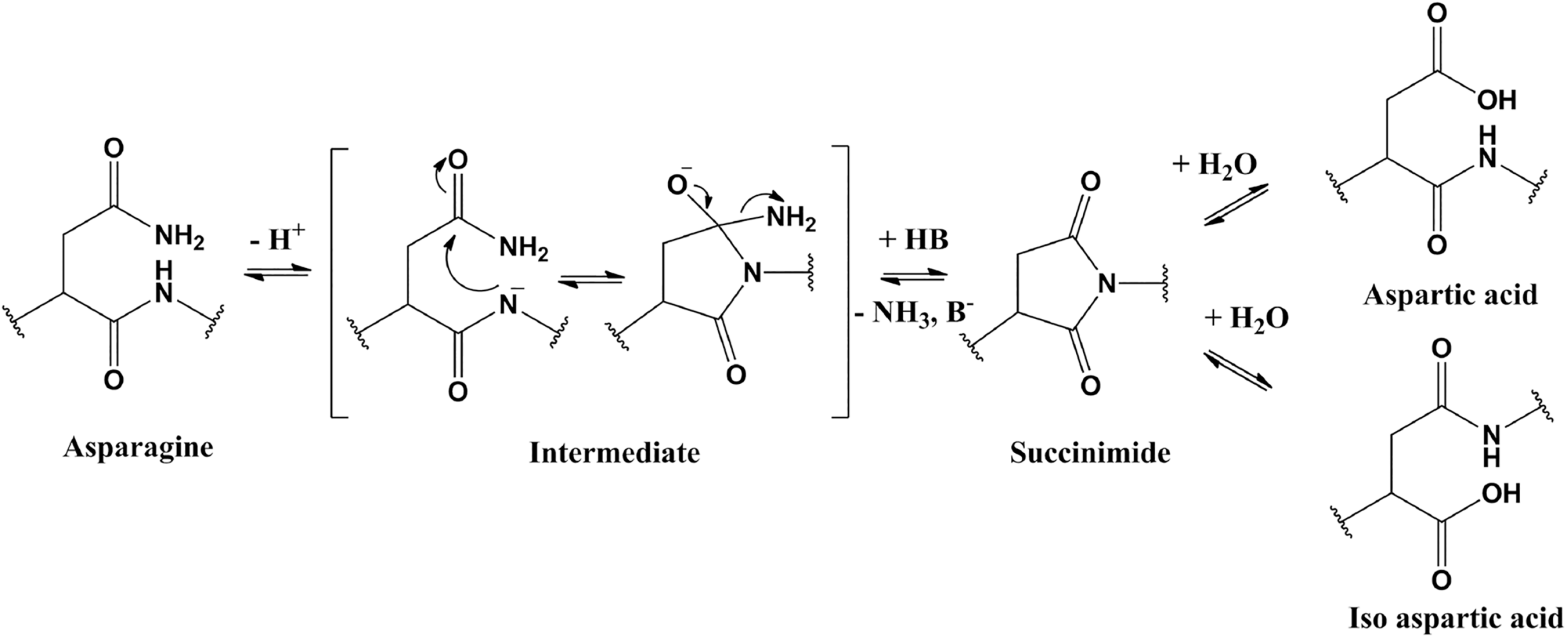
Chemical reaction of asparagine deamidation process.

Chemical stability is a major concern in the development of protein therapeutics due to its impact on both efficacy and safety. Protein “hotspots” are amino acid residues that are subject to various chemical modifications, including deamidation, isomerization, glycosylation, oxidation etc. Deamidation primarily happens on an asparagine (Asn) residue. Its C-terminus residue’s backbone nitrogen atom conducts a nucleophilic attack to the Asn’s side chain amide group carbon atom. An intermediate ring-closed succinimide residue is proposed to form. The succinimide residue then conducts fast hydrolysis to lead to the final product aspartic acid (Asp) or iso aspartic acid (IsoAsp) [1] (Fig 1). Therefore, the deamidation process causes an Asn to Asp / IsoAsp mutation. Glutamine (Gln) residue can also undergo the deamidation process. However, Gln deamidation happens at a much slower rate than Asn [2, 3], so it is a less concern. Deamidation of asparagine residues in biological pharmaceuticals is a major cause of degradation if the therapeutical proteins are not formulated and stored appropriately [4]. If deamidation occurs in the monoclonal antibody’s complementarity determining region (CDR), the antibody’s binding potency can be affected. Evaluating Asn deamidation liability is a very important step during the engineering process of therapeutical protein development.

Robinson *et al*. have been experimentally determined deamidation half-life of Asn containing penta peptides. The penta peptides have been designed by varying the N and C terminal residues adjacent to Asn which is at the center location of the peptide (sequence pattern: Gly-Xxx-Asn-Yyy-Gly) [5]. The data provide sequence-based foundation of Asn deamidation prediction. The same group then developed a “structure-based” deamidation prediction method with rule-based deamidation coefficient calculation [6]. Furthermore, Robinson *et al*. applied steric and catalytic effects of the Asn’s C-terminus residue type based on the penta peptides, whose deamidation half-life had been determined, to understand primary sequence control of deamidation as well as extend deamidation prediction to unnatural amino acid containing sequences [7]. The Houk group at UCLA and the Aviyente group at Bogaziçi University have used quantum mechanics method to study molecular details of deamidation process, separately [8–11]. Those quantum mechanics level studies helped us understand the reaction kinetics and the formation of succinimide intermediate better. However, they cannot be used to directly predict protein deamidation. Recently, Sydow *et al*. applied machine learning methods to predict monoclonal antibodies’ deamidation half-life [12]. Their lookahead-enabled single recursive partitioning tree model outperformed other machine learning methods and yielded 4.3% falsely assigned hotspots, which was much more accurate than the controlling sequence-based prediction false rate (43%). Lorenzo *et al*. developed NGOME, a sequence-derived secondary structure and intrinsic disorder based deamidation prediction method [13]. NGOME outperformed simple sequence-based method with area under the curve (AUC) value of 0.9640 v.s. 0.9270 for any deamidation and also outperformed simple sequence-based method with AUC value of 0.7051 v.s. 0.5 for the most challenging NG motif (amino acid asparagine followed by glycine) prediction.

Sequence-based prediction method simply identifies the NG motif to be liable to deamidation. NH, NS, and/or NT sequence motifs are also considered to be liable to deamidation based on their short penta peptide deamidation half-life. Sequence-based prediction method still dominates deamidation evaluation process in most pharmaceutical setups due to its convenience. However, the simple sequence-based method is less accurate and often causes over-engineering a protein. A more accurate prediction method would allow elimination or reduction of true deamidation liabilities as early as possible in the drug discovery process while avoid the over-engineering problem. In our work, we developed structure-based Asn deamidation prediction models with machine learning approaches. The prediction models were built to mine available experimental and high-resolution structural data of deamidated proteins. Experimentally measured deamidation half-life of Asn in penta-peptides as well as 3D structure-based properties, such as solvent exposure, crystallographic B-factors, local secondary structure and dihedral angles etc., were used to train prediction models with several machine learning algorithms. The prediction tools were cross-validated as well as tested with an external test data set. The application can make deamidation predictions to proteins but not limited to antibodies. The random forest (RF) model had high enrichment in ranking deamidated residues higher than non-deamidated residues while effectively eliminated false positive predictions, which is an advantage comparing to sequence-based prediction. Binary prediction of a “blind” test set by RF yielded 0.96 AUC value of receiver operating curve (ROC) as well as 0.65 Matthews correlation coefficient (MCC). In addition, we extensively discussed metrics being used to evaluate the performance of predicting unbalanced data sets such as the deamidation case. We also used feature selection to understand contributions of different descriptors on structural level.

## Methods

### Data set construction

The training data set consists of 194 asparagine (Asn) residues on 25 protein structures. Data were obtained from the following literatures [14–25]. NMR structures in [14] were replaced by high resolution crystal structures from the Protein Databank (PDB). The protein structure and sequences were obtained from PDB. The redundant structures and sequences from the same pdb structure were removed. The training data set contains different PDB entries of the same protein which have different structure features (e.g. closed and open conformations with and without substrate binding; different local structure etc.). The training data set also contains protein homologs from different species even with very high sequence similarity. Different structures from same or highly similar proteins with different structural features can reinforce the machine learning methods to pick up the subtle structural features instead of just the sequence. The prediction model is indeed more challenged with multiple crystal structures for the same protein, due to the variability of the structural descriptors. When a crystal structure has multiple chains, the first most complete chain to the relevant protein which contains the deamidation sites was used. “Clean Protein” function in BIOVIA Discovery Studio 4.0 was applied to all structures to correct problems related to structural disorder (structure with alternate conformations), protein residue connectivity and bond orders, missing side-chain or backbone atoms. It can also correct the enumeration of hydrogens according to the preferred hydrogen representation. We also constructed an external test data set, which was curated independently from the training set. All test proteins are distinct from the training set. It consists of 81 Asn residues on 3 high-resolution protein structures including an anthrax protective antigen, an angiogenin, and a glucoamylase [26–30]. The test set was prepared the same way as the training set. All structures have a resolution better than or equal to 2.5Å. There are additional deamidation data with lower resolution structures. Since B-factors, which are sensitive to resolution, are used as descriptors, we removed those data for this study. The deamidation data sets are binary (deamidation—positive or not deamidation—negative). In the training data set, there are 28 positive and 166 negative data. In the test data set, there are 5 positive and 76 negative data (Table 1). The training and test data sets are available in S1 Table.

**Table 1:** Data set construction.

Data sets	Number of proteins	Number of Asn residues	Deamidated Asn	Non-deamidated Asn
Training set	25	194	28	166
Test set	3	81	5	76

### Descriptor set

Our novel descriptor set is a combination of one experimental measurement and a set of structural features. The experimental measured penta peptide deamidation half-life was obtained from Robinson *et al*. [5]. The half-life of Gly-Xxx-Asn-Yyy-Gly represents the deamidation rate of a penta peptide with the adjacent residues at N and C termini of Asn (N) varied by a combination of amino acids. Residue Xxx includes G, S, T, C, M, F, Y, D, E, H, K, R, A, L, V, I, W, and P. There were no data for residues Xxx = N and Xxx = Q. In these 2 cases, we used Xxx = G data since the Xxx residue does not affect the deamidation half-life much. Residue Yyy includes G, H, S, A, D, T, C, K, M, E, R, F, Y, W, L, V, and I. There were no data for residues Yyy = N, Yyy = Q, and Yyy = P. In the case of Yyy = P, we manually assigned 999 to deamidation half-life since the nitrogen atom on proline is not able to perform nucleophilic attack to Asn side chain carbonyl carbon atom under normal conditions. In the case of Yyy = N (Gly-Xxx-Asn-Asn-Gly), we can shift one amino acid downward and use Xxx = N case. We cannot make prediction of the Yyy = Q case.

**Fig 2:**
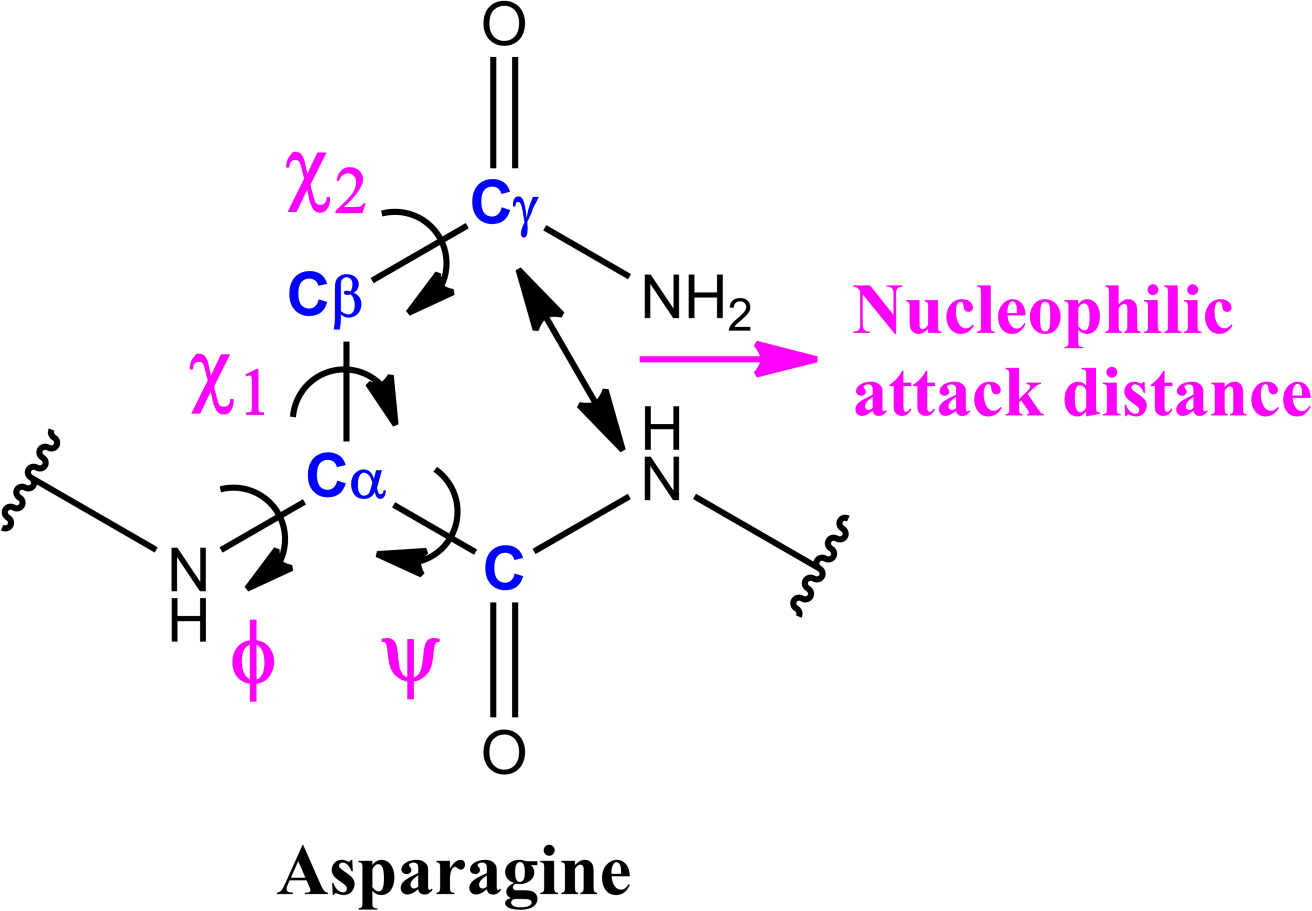
The structure-based descriptor set. The set of descriptors includes the nucleophilic attack C-N distance, normalized crystallographic B- factors at C, Cα, Cβ, and Cγ atoms (blue) of the Asn residue, and torsion angles Phi (φ), Psi (ψ), Chi1 (χ_1_) and Chi2 (χ_2_) (magenta).

To compensate the sequence-based penta peptide deamidation half-life descriptors, we developed a set of structure-based descriptors. The nucleophilic attack distance between Asn side chain carbonyl C atom and its C-term residue main chain amino N atom (Fig 2) represents the basic requirement of the chemical transformation to intermediates. Normalized crystallographic B- factors at C, Cα, Cβ, and Cγ atoms of the Asn residue (Fig 2) represent the flexibility at Asn local region. The B-factors were normalized by obtaining the standard score (z-score) against all atoms in a certain crystal structure [31]. Z-score = (x-μ)/σ, where μ and σ are the average and standard deviation, respectively, of all atoms’ B-factors in a pdb structure. B-factor normalization provides relative atomic flexibility to a certain protein. Asn residue and side chain percentage solvent accessibilities (PSA: Percent Solvent Accessibility and PSSA: Percent Sidechain Solvent Accessibility) were calculated to represent its solvent exposure. BIOVIA Discovery Studio 4.5 was used to calculate PSA and PSSA. Backbone torsion angles–Phi (φ) and Psi (ψ) and side chain torsion angles Chi1 (χ_1_) and Chi2 (χ_2_) of the Asn residue (Fig 2) represent Asn conformation. We also included Asn local secondary structure which was quantified as the following: alpha helix = 1, beta sheet = 2, coil = 3, and turn = 4. The local secondary structure was assigned with BIOVIA Discovery Studio 4.0. In total, 13 descriptors were developed for building the prediction model (Table 2).

**Table 2:** Different types of descriptors that were developed for building deamidation prediction models.

Experimental measurement (days)	Penta-peptide deamidation half-life
**Conformations: Asn torsions (degrees)**	Backbone torsion Phi (φ)
	Backbone torsion Psi (ψ)
	Sidechain torsion Chi1 (χ_1_)
	Sidechain torsion Chi2 (χ_2_)
**Conformations**	Asn local secondary structure
**Flexibility: Normalized B-factors for key carbons on Asn**	Backbone carbonyl C
	Backbone C alpha (C_α_)
	Sidechain C beta (C_β_)
	Sidechain C gamma (C_γ_)
**Solvent exposure**	Percent Solvent Accessibility (PSA)
	Percent Sidechain Solvent Accessibility (PSSA)
**Reaction coordinate (Å)**	Nucleophilic attack C-N distance

### Statistics algorithms

Different statistics algorithms have different advantages which are suitable for specific training and prediction purpose. To obtain high performance models for Asn deamidation prediction purpose, we tested several statistics algorithms. Five supervised machine learning algorithms: support vector machine (SVM), random forests (RF), naïve Bayes classifier (NBC), K nearest neighbor (KNN), and artificial neural network (ANN) and a regression algorithm partial least squares (PLS) were applied to model building. All statistics algorithms were implemented with the Caret package (v 6.0–35) [32] in the R project for statistical computing (v 3.1.1). The R script is available in S1 File.

The method of support vector machines (SVM) was developed by Vladimir N. Vapnik at AT&T Bell Labs originally for discriminative classification to solve handwriting recognition problems [33]. It’s capable to explore subtle patterns in a noisy data set by applying kernel functions and soft margins. This kernel based SVM is very powerful to make predictions by projecting the data to a higher dimensional feature space by a kernel function. However, using the kernel function may introduce overfitting problem. The random forests (RF) method was developed by Leo Breiman of UC Berkeley [34]. Advantages of RF include the ability to establish interpretable models, accurate predictive results, resistant to overfitting problems, and fast training process. Naïve Bayes classifier (NBC) is based on Bayes’ theorem [35]. It can only be applied for classification. NBC requires only a small amount of training data to estimate the parameters necessary for classification and can be scaled very well to very large data sets. NBC has a little difficulty with noisy or missing data. K nearest neighbor (KNN) is a method for classifying objects based on closest training examples in the feature space (feature similarity clustering) [36]. KNN is one of the simplest machine learning algorithms. The interpretable algorithm has simple implementation in which only one parameter–K needs to be tuned. One disadvantage of the method is that it’s computationally intensive. Artificial neural network (ANN) is a mathematical model that is inspired by the structure and functional aspects of biological neural networks [37]. ANN is one commonly used artificial intelligent (AI) tool and able to learn from training data. When an element of the neural network fails, it can continue without any problem by its parallel nature. ANN requires a large diversity of training set in real-world operation. Partial least squares (PLS) is one of the most commonly used regression tools in bioinformatics and cheminformatics [38]. It’s an extension of the multiple linear regression method. PLS can reduce many factors to a few latent factors thus avoids overfitting problem.

### Model building, validation and testing

Before training, the data were centered and scaled by using a preprocess function in each of the training algorithms. We built binary classification models to predict a residue can be deamidated or not. The model also output probabilities for classification, which can be used to rank the deamidation tendency. The Caret package automatically tuned the parameters in these models by grid search. Ten-fold cross validation was carried out by using the same training set. The accuracy of the 10-fold cross validation was used for evaluating different statistics algorithms.

To rigorously evaluate the modeling performance for generally predicting real data, a “blind” test was carried out for each model. Those deamidation data were independent from the training data set. The accuracy from this test process was used for checking whether the models were overfitted. In addition to prediction accuracy, ROC was plotted and AUC value was calculated for each model to evaluate the prediction performance. ROC plots correlation between True Positive Rate (TPR) and False Positive Rate (FPR). They are defined as the following:

$$True\;Positive\;Rate\;(Recall)=\frac{TP}{TP+FN}$$

$$False\;Positive\;Rate=\frac{FP}{FP+TN}$$

Confusion matrix: True Positive (TP); False Positive (FP); True Negative (TN); and False Negative (FN) was calculated in order to compute additional statistical metrics. Accuracy is the ratio between corrected predicted cases and all cases. It can only provide a very general evaluation of the classifier’s performance. Accuracy lacks the enrichment of the model. AUC value represents the enrichment of the prediction based on true positive rate (recall) vs false positive rate on the ROC plot. Thus the AUC value is better in evaluating prediction performance than accuracy. Since our data sets were unbalanced toward to negatives, positive prediction (predicting deamidation case) was more challenging than negative prediction. Given the limitation of AUC value for classification prediction of unbalanced data set [39], we also analyzed precision, recall (as known as TPR), and specificity metrics to further evaluate the positive prediction performance in comparing to negative prediction performance. Finally, we calculated Matthews correlation coefficient (MCC) [40] as a single metrics to capture full confusion matrix.

$$Precision=\frac{TP}{TP+FP}$$

$$Specificity=\frac{TN}{FP+TN}$$

$$MCC=\frac{TP*TN-FP*FN}{\sqrt{\left(TP+FP)(TP+FN)(TN+FP)(TN+FN\right)}}$$

### Feature selection

In order to connect statistics modeling results to the biophysical and structural information, feature selection was performed with recursive feature elimination (RFE) method from the Caret package. RFE evaluates critical descriptors which contribute most to the prediction models. The high impact descriptors can help protein scientists to better design protein mutants and construct screening libraries based on understanding the protein properties on structural and biophysical levels.

## Results and discussion

### Data set construction

Our training data set contains 25 proteins and 194 Asn residues which have risk to deamidation. Among the 194 Asn residues, 28 were experimentally measured to be deamidated. And the rest of 166 residues were stable. The independent test set contains 3 proteins and 81 Asn residues. Among the 81 Asn residues, 5 were experimentally measured to be deamidated. And the rest of 76 residues were stable (Table 1). All proteins have high-resolution x-ray crystal structures for structure-based prediction. Due to the nature of deamidation which is not frequently observed, and the availability of high resolution crystal structure, the data sets are both small and unbalanced. However, the training data is similar to the test and target data in terms of proportions of the two binary outcomes. Even though the size of data sets are limited, the protein structures are reasonably diversified, containing a wide variety of protein classes as defined in Structural Classification of Proteins database (SCOP) [41]. In this work, we demonstrated a predictive deamidation model can still be obtained with such a small training data set by well developing a descriptor set tailored to deamidation mechanism.

### Training binary deamidation prediction models and cross validation

The binary deamidation prediction models were trained with 6 different statistics algorithms. Ten-fold cross validation (CV) was conducted to evaluate the overall training performance of different algorithms (Table 3). All models had reasonable overall CV accuracy (greater than 0.8). Random Forest (RF) outperformed others and reached 0.9 accuracy. The RF algorithm is based on binary decision trees. We focused on the RF model in the further testing study.

**Table 3:** Cross validation of binary deamidation prediction models.

Methods	SVM	RF	NBC	KNN	ANN	PLS
CV Accuracy	0.86	**0.90**	0.83	0.86	0.86	0.86

### Testing prediction models with an external test set

**Fig 3:**
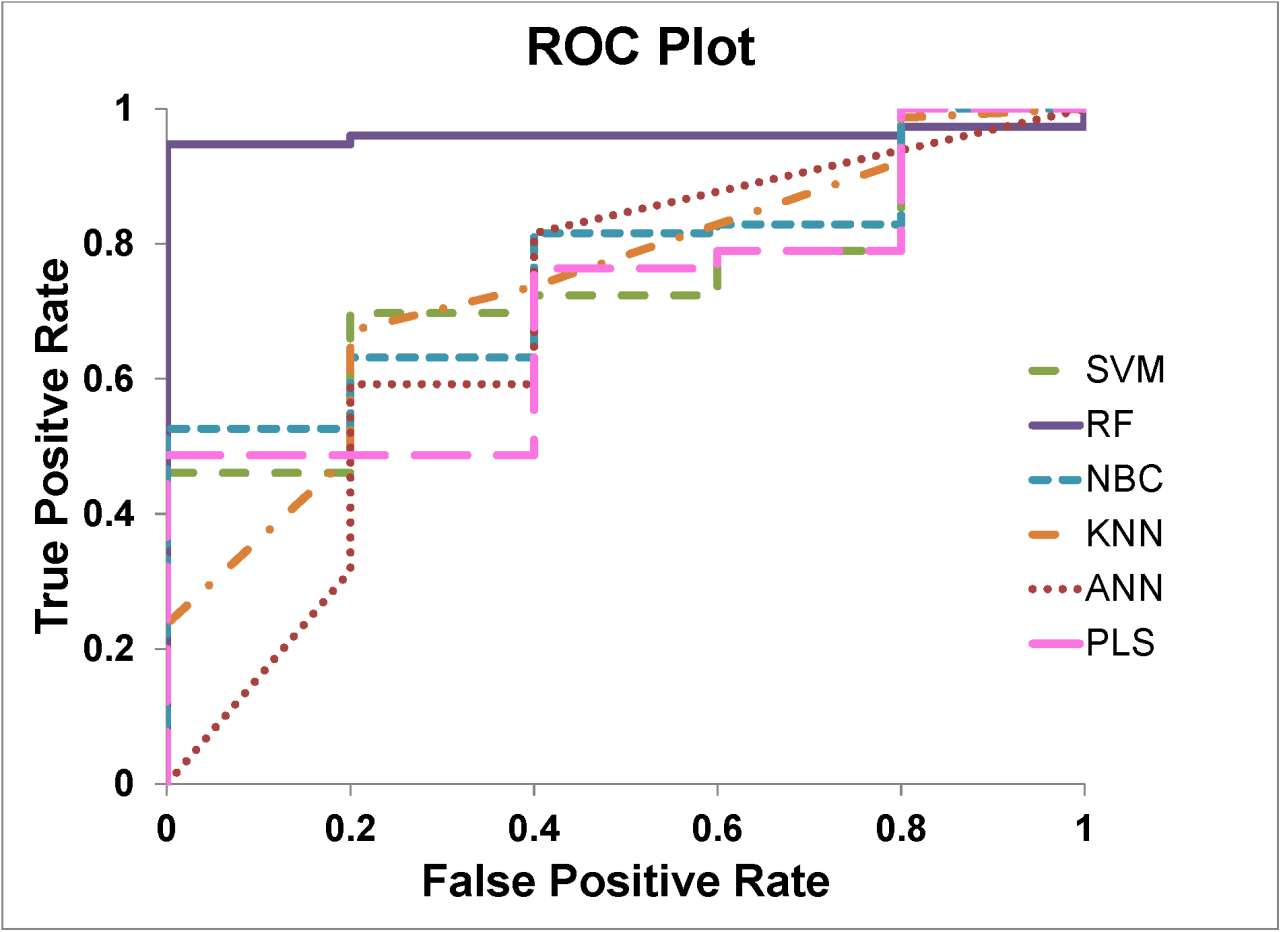
ROC plots for external test set predictions with different methods. The RF method (purple solid line) outperformed the rests.

To rigorously test the prediction models, we used an independent test set for blind prediction exercise. In the test process, besides to the overall accuracy, we used additional performance measurements to further evaluate the prediction models. True positive and negative counts are cases of correctly predicted to be deamidation and non-deamidation, respectively. False positive and negative counts are cases of wrongly predicted to be deamidation and non-deamidation, respectively. AUC value of receiver operating characteristic (ROC) plot was used to evaluate the enrichment of positive prediction results (Fig 3).

Table 4 shows the test results. Comparing between CV and the blind test, the overall accuracy was generally increased (except for NBC). This comparison demonstrated that most models were not overfitted. Even though the overall accuracy was high in most models, the enrichment measurement by using the AUC value revealed difference between models. The RF model outperformed others again in the testing case. S1 Table reported the prediction details of the RF model with deamidation probability. By ranking the probability, all 5 deamidated Asn were enriched with highly ranked probabilities.

**Table 4:** Blind test of binary deamidation prediction models.

Methods	SVM	RF	NBC	KNN	ANN	PLS
Accuracy	0.94	**0.95**	0.75	0.94	0.94	0.95
True Positive	0	**4**	3	0	0	1
True Negative	76	**73**	58	76	76	76
False Positive	0	**3**	18	0	0	0
False Negative	5	**1**	2	5	5	4
**AUC**	0.73	**0.96**	0.76	0.74	0.69	0.71
**Recall (TPR)**	0	**0.80**	0.60	0	0	0.20
**Specificity**	1	**0.96**	0.76	1	1	1
**Precision**	-	**0.57**	0.14	-	-	1
**MCC**	0	**0.65**	0.20	0	0	0.44

Since the deamidation data sets are unbalanced toward the negative case, it is necessary to further evaluate the performance in predicting positive and negative cases separately. Recall was used to measure how accurate the model can correctly predict deamidated Asn (positive cases) among all experimentally measured deamidated Asn, while specificity was used to measure how accurate the model can correctly predict non-deamidated Asn (negative cases) among all experimentally measured non-deamidated Asn. We observed more challenges in positive prediction than negative prediction when comparing recall and specificity. All methods had specificity greater than 0.75 for predicting negatives. But the best recall was 0.80 from the RF model by which 80% of all measured deamidated Asn could be correctly predicted. SVM, ANN, and KNN simply predicted all test cases to be negative. So they reached an extreme of 0 for recall and 1 for specificity. Such prediction performance is not acceptable but was rather influenced by artifact from the unbalanced training data. However, simply using accuracy cannot reveal this performance issue. When considering positive and negative prediction performance separately, RF still outperformed other models.

### Investigation of NG-motif based deamidation prediction and comparison to another sequence-based prediction

**Fig 4:**
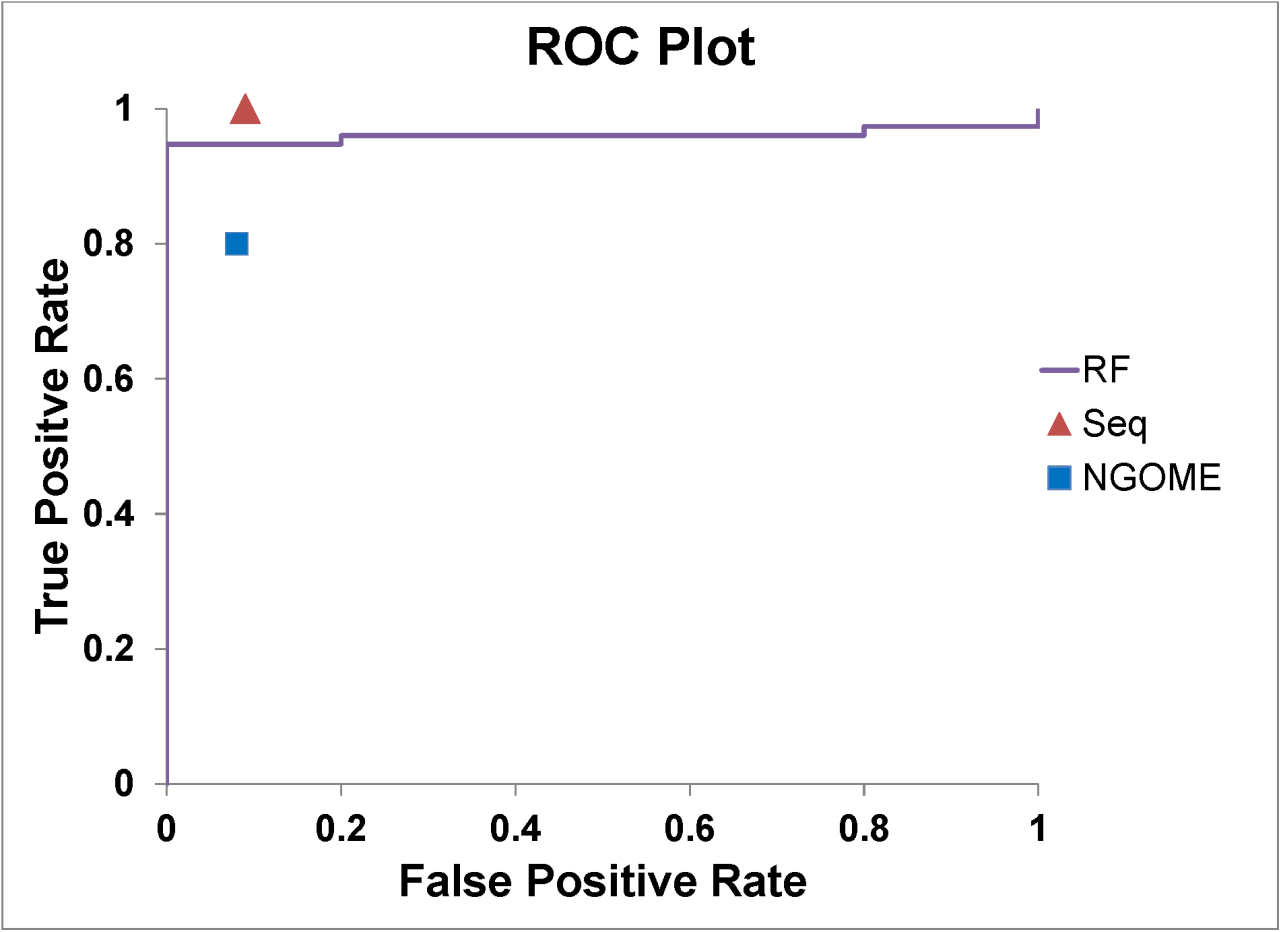
Performance comparison between NG-motif, NGOME and our structure-based methods. NG-motif and NGOME prediction performances were represented by plotting the TPR v.s. FPR points (red triangle for NG-motif and blue square for NGOME) on the ROC of the RF method (purple line).

The penta-peptide deamidation measurement showed that the NG motif subjected the highest risk of deamidation. Actually, some sequence-based deamidation tools just simply flag all NG motifs to be deamidated. In reality, not all NG motifs are liable to deamidation. We tried to use our structure-based prediction method for more accurately predicting non-deamidated NG motifs, which are the most challenging in all deamidation prediction cases. Correctly predicting non-deamidated NG motifs is also very important in designing mutation screening libraries to effectively narrow down the library size and avoid over-engineering proteins. In addition, we compared our structure-based method with another published sequence-based method NGOME [13]. Same test set was use for NG-motif and NGOME methods. The NG-motif method simply predicted all NG-motifs to be deamidated and other asparagine residues not being deamidated. We used the NGOME web service with their default parameters to conduct sequence-based prediction. The RF method from our work was used for comparison. Table 5 showed comparison between the 3 methods. Fig 4 highlighted the NG-motif and NGOME performance on the ROC plot of the RF method. Comparing all three methods, our structure-based method performed slightly better than the NG-motif method, and both methods performed better than NGOME, which had a higher FP comparing to our RF method and a higher FN comparing to NG-motif method. NG-motif method had better recall but sacrificed specificity and precision comparing to our RF method. Increased false positive prediction causes over-engineering of protein which can lead to stability and activity issues.

**Table 5:** Comparison between NG-motif, NGOME, and our structure-based prediction methods.

Methods	NG-motif	NGOME	Structure-based (RF)
Accuracy	0.91	0.91	**0.95**
True Positive	5	4	**4**
True Negative	69	70	**73**
False Positive	7	6	**3**
False Negative	0	1	**1**
**Recall (TPR)**	1	0.80	**0.80**
**Specificity**	0.91	0.92	**0.96**
**Precision**	0.42	0.40	**0.57**
**MCC**	0.62	0.53	**0.65**

Our best model, RF method, correctly predicted 4 out of 7 non-deamidated NG motifs (S1 Table). This accuracy is acceptable but not very high. Two directions may improve prediction accuracy of the non-deamidated NG motif: 1. Develop more sophisticated descriptors by understanding the difference between deamidated and non-deamidated NG motifs. 2. Enrich the data set to increase signal to noise ratio. On the other hand, SVM, ANN, and KNN correctly predicted all 7 non-deamidated NG motifs by simply predicting all residues in the test set (including the 5 deamidated NG motifs) to be negatives (Table 4). So in term of the overall performance, SVM, ANN, and KNN are no better than the NG-motif method.

### Feature selection and understanding protein deamidation on structure level

To connect the statistics prediction with biophysical and structural information of the models, feature selection was carried out with recursive feature elimination (RFE) in the Caret package. RFE ranks the importance of the descriptors by comparing their weight contribution in a model. We used the best performed RF model to conduct feature selection. The experimentally measured penta-peptide deamidation half-life ranked the highest among all descriptors with a heavy weight (Table 6). It demonstrated the importance of this experimentally measured property in the prediction model. Although most of other weights are similar, we did observe some differences which made scientific sense. Asn torsion angles as well as normalized B-factors played very important roles in the deamidation model. Torsion angle describes detailed conformation which significantly influences the position of the Asn side chain carbonyl carbon atom and its C-term residue’s backbone amino nitrogen atom, the key atoms in the deamidation chemical reaction. Among the 2 backbone torsions, psi is more important than phi. Psi is the torsion close to the C-term residue of Asn, while phi is the torsion close to the N-term residue of Asn, (Fig 2). This observation is consistent to the evidence that the C-term residue of Asn has more influence to deamidation than Asn’s N-term residue. The side chain torsion chi2 is more important than chi1. Chi2 is closer to the nucleophilic attacked carbonyl than chi1. Backbone torsions are generally more important that the side chain torsions. This indicates that the backbone conformation is more important to influence deamidation than the side chain conformation.

**Table 6:** Features (descriptors) ranking by RFE (the RF model).

Features	Weight
Half_life	100
norm_B_factor_CB	17.629
Psi	16.434
Chi2	16.356
Phi	14.112
norm_B_factor_CA	12.762
Chi1	8.849
norm_B_factor_C	8.619
norm_B_factor_CG	7.600
PSA	6.842
PSSA	5.384
attack_distance	5.075
secondary_structure	0

The normalize B-factors provide the flexibility measurement which is very useful information on top of the static coordinate from the X-ray crystallographic data. In order to conduct deamidation reaction, we hypothesized that the Asn-Yyy residues should be flexible enough to allow the reactive carbon and nitrogen atoms to approach to a feasible distance for nucleophilic attack as well as avoid clashes between newly formed succinimide intermediate and its surrounding environment. We observed that the CB’s B-factor has a significant higher contribution to the model than other B-factors. The next important B-factor is CA’s. CB is generally more flexible than backbone C and CA atoms. Both CA and CB flexibility can lead to higher flexibility on CG since they are covalently linked. And CG is the end carbon atom out of the 4.

The PSA and PSSA were ranked slightly lower than the torsional and flexibility descriptors but with not so different weights. Surface residues have relatively higher flexibility than that of buried residues. In addition, surface residues are more accessible to water, which plays important role in the deamidation reaction. All of these rankings did make structural biology sense. To our surprise, the nucleophilic attack C-N distance did not rank high. But this feature has a direct connection to deamidation reaction. Note that we obtained the nucleophilic attack the carbon and the nitrogen atoms (C-N) distance by using the static coordinates from X-ray crystallography, which did not necessarily represent the actual dynamics of the C-N distance in solution. Using the C-N distance from ensemble structures by molecule dynamics simulations of these proteins would make more sense.

Purely sequence-based prediction by using only experimentally measured deamidation half-life as a single descriptor would derive a model which predicts all NG motifs to be deamidated (the NG-motif method). With additional structure-based descriptors, some non-deamidated NG can be identified. Moreover, structural descriptors help protein engineers to better understand deamidation at the molecular level and provide guidance in stable protein design.

### Evaluating models with unbalanced data set

There are many metrics which can be used to evaluate the performance of a prediction model. Accuracy and AUC value are the most commonly used metrics for this purpose. Accuracy can only provide a very general evaluation of the classifier’s performance without enrichment information. However, AUC value describes the enrichment of the prediction based on true positive rate (recall) vs false positive rate on the ROC plot (Fig 4). The enrichment information can be useful to evaluate unbalanced data set. For example, both PLS and RF methods had the same 0.95 overall prediction accuracy, since they all made 4 false predictions (Table 4). However, the RF method was considered as a much better performed model than PLS because it more effectively predicted the positives (4 out of 5) which was a much smaller portion comparing the negatives in the data set. While the PLS method only correctly predicted 1 out of 5 positive cases. The AUC values of RF (0.96) and PLS (0.71) reflected the difference.

On the other hand, AUC value cannot always provide unbiased evaluation to classifiers with unbalanced dataset as suggested by Lever et al [39]. For unbalanced data sets, using recall, specificity, and precision can also be very effective to evaluate the true performance of the model. These metrics are all calculated based on the confusion matrix. In our example, both training and test data sets were unbalanced toward negatives. There are much more non-deamidated Asn than deamidated Asn in proteins. So in our models, it is very easy to reach high specificity across the board (Table 4), but there is a challenge to reach high recall. In an extreme case, if the method blindly predicted all Asn to be negatives as in SVM, ANN, and KNN, the specificity reached 1, and the recall was 0. These three methods perfectly predicted negatives but failed to correctly predict any positives. Practically speaking, we care more of accurately predicting deamidated residues. As the performance of the models, recall would be more focused comparing to specificity. So SVM, ANN, and KNN were the three worst models. On the other hand, the recall of the RF model reached 0.80, which was considered to be satisfied.

Precision is to evaluate the positive prediction efficiency. It is used to measure how efficient of the positive prediction (among all predicted deamidation cases, the portion that is correctly predicted). It should not be confused with recall. The major diffidence between the two metrics is that precision considers the success of positive prediction among all predicted positives. Recall considers the success of positive prediction among all *experimentally measured* positives (positive as a fact). For our purpose, balancing both high recall and precision is important since we care how accurate the deamidated residues can be predicted (recall) as well as how efficient of the prediction to avoid many false positives (precision). In the PLS model, the precision was 1. This model only predicted one Asn to be positive. And that one Asn was correctly predicted (Table 4). However, the rest of 4 measured positive cases were not correctly predicted. So, its recall was only 0.2. Therefore, although PLS had a perfect precision, it was not considered to be a good model due to the low recall. In the above discussed sequence-based method, all NG motifs were simple flagged as positives. Even though the recall was higher than the RF method (1 vs 0.8, Table 5), there were more false positives so that the precision dropped to 0.42, comparing to 0.57 in the RF model. Therefore, the sequence-based method was also not an optimized method.

Moreover, Matthews correlation coefficient (MCC) value captures information of the whole confusion matrix. It can be used to evaluate classifiers derived from unbalanced data sets as a single value. MCC ranges from -1 which, reflects that the prediction is always wrong, to 1, which reflects that the prediction is always correct. An MCC of 0 reflects a random prediction [39, 40]. In our test cases, RF has the highest MCC value of 0.65. SVM, ANN, and KNN have the worst MCC of 0. These three methods made random predictions since they predicted all Asn residues to be non-deamidated (negative).

In summary, when assessing the performance of predictions with unbalanced data sets, the purpose of prediction should be considered upfront. (Whether true positive or true negative prediction is considered to be more important and which part is the minor portion?) Then the right metrics (recall, specificity, precision, or MCC) can be selected to represent the performance.

### Application to aspartic acid isomerization

The similar reaction can happen to Asp residue and transforms it to IsoAsp. Such reaction makes Asp being considered as a protein hotspot of isomerization. Asp isomerization has been reported on antibodies by multiple papers [42–44]. Comparing to Asn deamidation, Asp isomerization has a higher rate at low pH (< 5.5) [1]. So when protein is formulated at low pH condition, Asp isomerization becomes a concern. Unlike Asn deamidation, Asp isomerization is more challenging to be detected by mass spectrometry due to the same molecular mass of the product IsoAsp comparing to Asp. Thus, accurate prediction of Asp isomerization is even more important than Asn deamidation prediction. Our Asn deamidation prediction method can be applied to Asp isomerization prediction. The descriptor set is capable to represent isomerization. More accurate results can be obtained with protein isomerization specific data set, which is more difficult to obtain due to the above mentioned measurement challenge. Sharma *et al*. reported an antibody isomerization prediction method based on logistic regression with consideration of solvent exposure and flexibility of the Asp residue [45]. Their prediction to the liable motifs DD, DG, DS, and DT performed pretty well with only one reported false negative.

## Conclusions

The machine learning models, using structure-based descriptors, described in this work for deamidation prediction achieved improved accuracy compared to existing methods. The application can make deamidation predictions to proteins but not limited to antibodies. Compared to sequence-based methods, structure-based methods provide insights to better understanding the molecular basis of deamidation event. Enriching the training data set to contain more diversified protein deamidation data would further increase the prediction accuracy. Using machine learning methods to predict structure-function relationship in protein engineering faces lots of challenges. Proteins have highly diversified structures being encoded by their primary sequences. And proteins undergo concerted dynamics in order to conduct their functions. In this work, the descriptor set for deamidation prediction has been well developed in connection to chemical reactions. The set includes an experimental measurement as well as structural features. However, there could be improvements when the dynamics of the protein, which can be investigated by molecular dynamics simulations, is considered. Under the circumstance of the imperfect training data set and descriptor set, the statistical machine learning algorithms play important roles in removing the noise, and amplifying the signal of the data. Theoretically speaking, when perfect descriptors can be obtained, the very simple statistical algorithm should be good enough to construct a predictive model without overfitting the data. While in reality, we always balance the three key components: the training data set, the descriptor set, and statistical algorithms, in a prediction modeling process to optimize prediction performance. Finally, since protein engineering data sets are often noisy and unbalanced, it’s critical to select proper statistical metrics for performance evaluation.

## Supporting information

S1 Table Data sets being used for training and testing as well as the RF model results. (XLSX)

S1 File R script for training and test models. (TXT)
